# Supplementary material for: Klf4 methylated by Prmt1 restrains the commitment of primitive endoderm
Source: Nucleic Acids Res. 2022 Feb 7;50(4):2005–18. doi: 10.1093/nar/gkac054 (PMC8887470; doi:10.1093/nar/gkac054)
Supplement: gkac054_Supplemental_File [file gkac054_supplemental_file.pdf]

Supplemental information

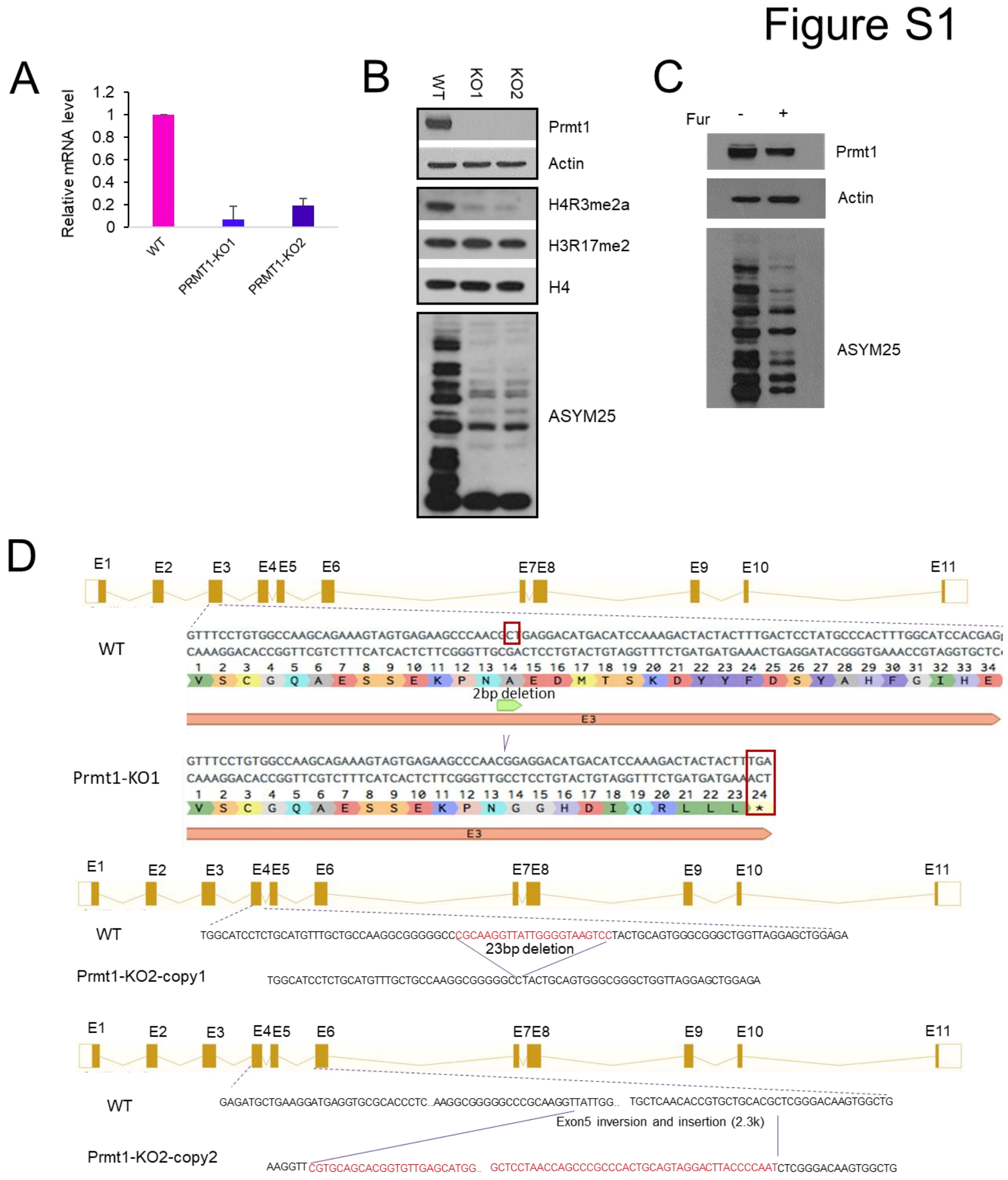

Figure S1. The effect of Prmt1-KO cells.

(**A**) The mRNA levels of Prmt1 decreased in Prmt1-KO cells. (**B**) Western blotting with anti-Prmt1, anti-Actin, anti-H4R3me2a, anti-H3R17me, anti-H4, and anti-ASYM25 in E14 ES cells (WT) and Prmt1 KO cells. (**C**) Western blotting with anti-Prmt1, anti-Actin, and anti-ASYM25 in Fur treated (+) or untreated (-) E14 cells. (**D**) The disruption of the Prmt1 genomic sequence within the knockout cell lines (KO1 and KO2).

Figure S2

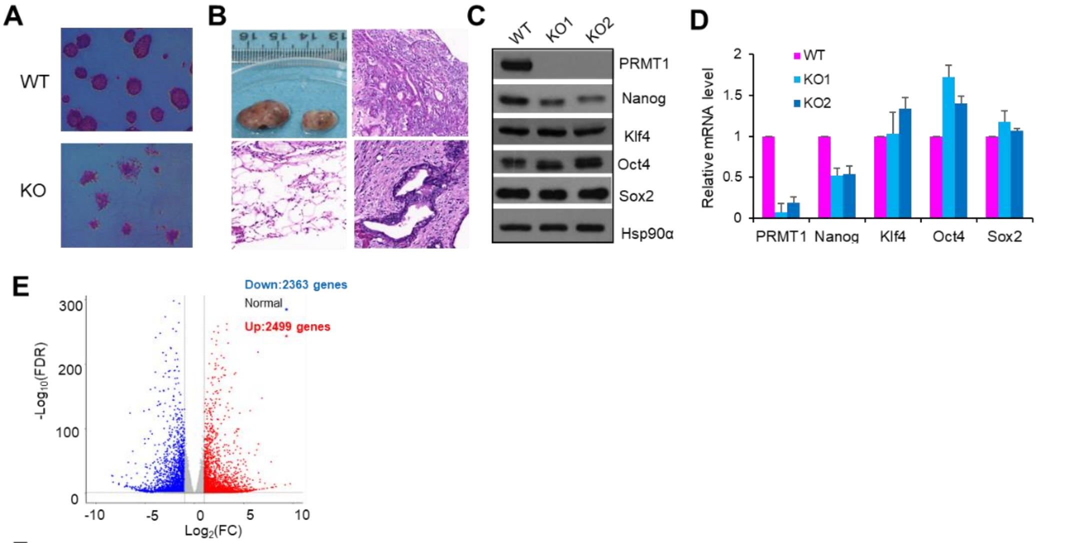

F

Term	Genes	Count	%	P-Value	FDR
developmental process		306	24.5	1.1E-13	7.6E-13
biological adhesion		106	8.5	7.9E-10	2.8E-9
localization		262	20.9	5.5E-7	1.3E-6
locomotion		90	7.2	4.1E-6	7.1E-6
biological regulation		482	38.5	6.5E-6	9.1E-6
single-organism process		547	43.7	4.5E-5	5.3E-5
growth		63	5.0	5.9E-5	5.9E-5
regulation of biological process		457	36.5	7.1E-5	7.1E-5
multicellular organismal process		339	27.1	1.2E-4	1.2E-4
behavior		45	3.6	2.3E-4	2.3E-4
signaling		266	21.3	2.4E-4	2.4E-4
reproductive process		84	6.7	1.6E-3	1.6E-3
reproduction		84	6.7	1.7E-3	1.7E-3
immune system process		106	8.5	1.0E-2	1.0E-2
cellular component organization or biogenesis		240	19.2	4.0E-2	4.0E-2
response to stimulus		343	27.4	6.4E-2	6.4E-2
rhythmic process		20	1.6	8.0E-2	8.0E-2

Term	Genes	Count	%	P-Value	FDR
single-organism process		426	39.6	1.8E-4	3.4E-3
reproductive process		67	6.2	2.6E-3	1.6E-2
reproduction		67	6.2	2.7E-3	1.6E-2
immune system process		88	8.2	3.3E-3	1.6E-2
localization		183	17.0	9.9E-3	3.8E-2
locomotion		56	5.2	3.2E-2	1.0E-1
developmental process		187	17.4	4.2E-2	1.2E-1

Figure S2. RNA-seq analysis of WT and Prmt1-KO cells.

(**A**) Alkaline phosphatase staining of WT and Prmt1 KO cells. (**B**) Hematoxylin and eosin staining of teratoma derived from PRMT1 KO mESCs. (**C** and **D**) Expression of the indicated genes by using Western blot (C) and RT-qPCR (D). (**E**) Differential genes expression analysis of bulk RNA-seq data of WT and Prmt1-KO cells. (**F**) Enriched GO terms from DAVID 6.8 for Prmt1upregulated genes (upper panels) and downregulated genes (lower panels) according to the bulk RNA-Seq data of WT and Prmt1 KO cells.

Figure S3

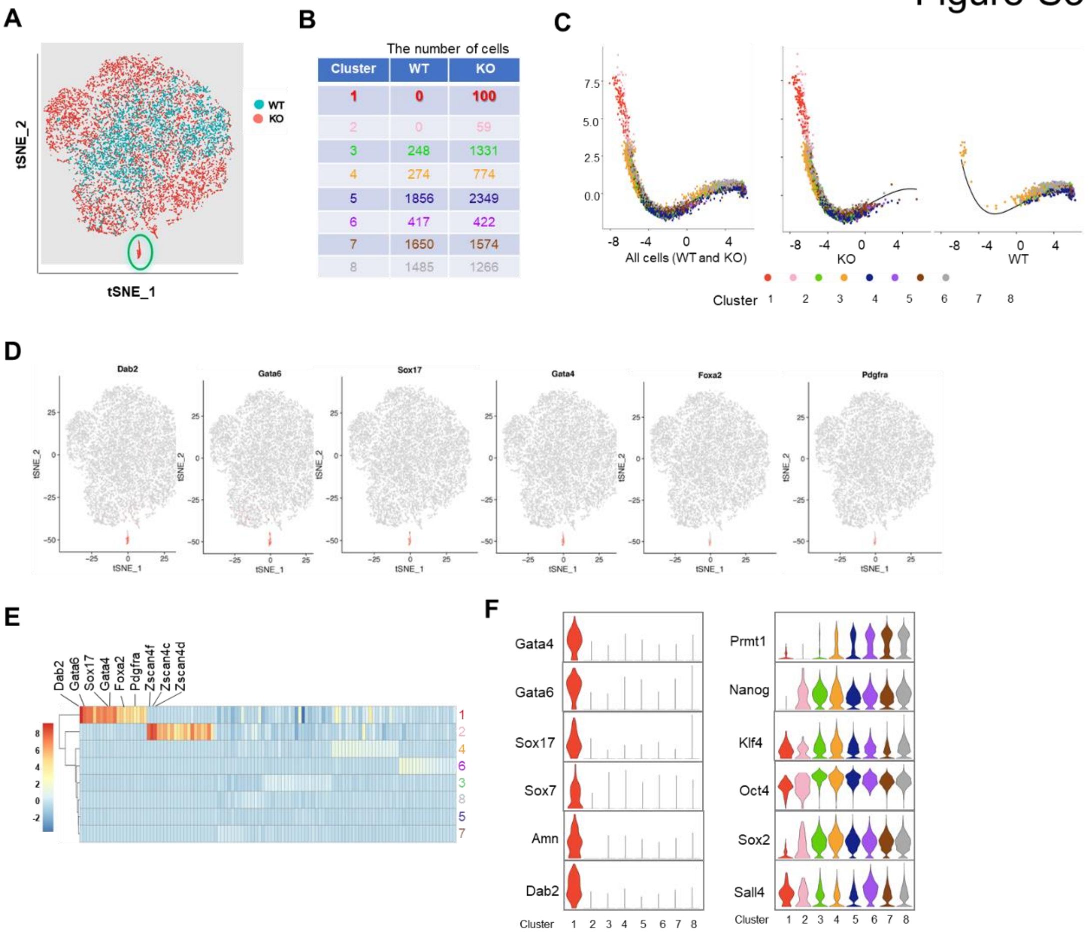

Figure S3. The analysis of sc-RNA seq.

(**A**) tSNE of 13,805 cells by scRNA-seq, include 5930 WT cells and 7875 Prmt1 KO cells which is related in Figure 4D. (**B**)Eight transcriptionally distinct clusters were identified (clusters 1-8) by unsupervised classification. Number and origin of cells in each cluster. (**C**) Principal component analysis (PCA) based on pseudotime showed that cluster 1 was distant from the other clusters. (**D**) Heatmap displaying the expression of marker genes in each cluster. (**E**) Cluster 1 and 2 are marked with indicated PrE genes and 2C-like genes. (**F**) Violin map showed that PrE genes are significantly and specifically high expression in cluster 1.

Figure S4

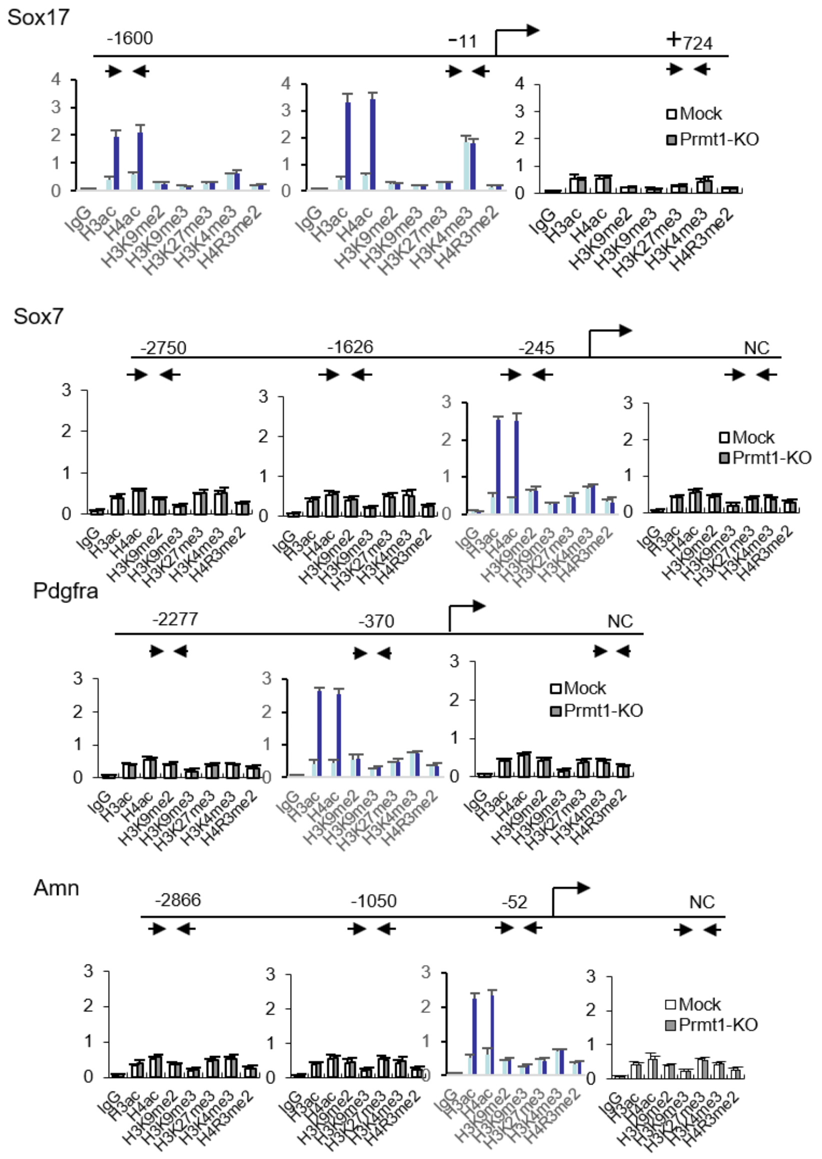

Figure S4. ChIP-qPCR analysis of histone modifications in upstream region of other PrE genes (Sox17, Sox7, Pdgfra, and Amn) in WT and Prmt1-KO cells.

Figure S5

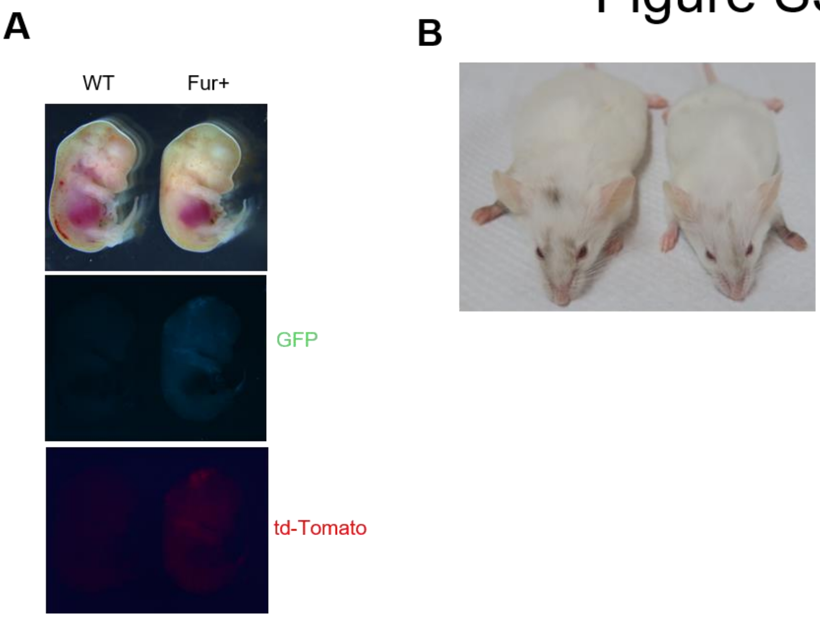

Figure S5. The chimeric embryos at E12.5 (A) and mice after birth (B) showed that the Fur-treated ES cells.

Table S1. The potential off-targeting locus were amplified by PCR and then sequenced, showing no mutant.

off target sequence	loci in genome	forward primer sequence	loci in genome	reverse primer sequence	loci in genome	length	Mutant
TAGTTAGAACCCCAACACTGAGG	chr6:+67353807	CCAACCCTGCTCAACCATCT	chr6:+67353436	AGCTTCATTACCCCAGCACC	chr6:+67354003	567	None
TAGTGAGAAGCCTAACACTGAGG	chr18:+4484501	GTGGCCTTGCAAATCAGCTC	chr18:+4484239	CCTACTCCACCTTGCCCTTG	chr18:+4484832	593	None
TAGGGAGAAACCCATCGCTGCAG	chr16:-72356018	TAAAAACCCATGGCACTCCC	chr16:-72356290	CATGTGCCCTTTTCCGTCC	chr16:-72355895	395	None
CAGTAAGTAGCCCAACTCTGAGG	chr3:+65369957	TCCTCCTGTTACCCTCCCTG	chr3:+65369659	GAACACTCACGGCTTCCTCA	chr3:+65370340	681	None
GGCTGAGAAGCCCAACTCTGTAG	chr12:+5140528	GCTTACTCTGGTCACAACGC	chr12:+5140288	AGCAGAAGTGTGCGCTAGG	chr12:+5140743	455	None
CACTAAGAAGCCCAACTCTGAGG	chr1:-5090179	TCAGAACCAAGCAGAGGCAG	chr1:-5090364	TGCGCCTCAGAGATGAGTTG	chr1:-5090019	345	None
TTGACAGAAGCCCAACCCTGCAG	chr7:-90034818	TCAGCCACATGAGATGCTGT	chr7:-90035173	GAATCCAAGGTGTGATGTGCT	chr7:-90034415	758	None
TAGTAAGAAGCTCAACACTGTAG	chr18:-42730599	TCACCGCAGAGGTTTCGTC	chr18:-42730825	CGCTGACAGTAGTGGTCCTATC	chr18:-42730457	368	None
TAATGAGCACCCCAACACTGGGG	chr8:-118620357	CCCCAGGTCAGAGTAGGTGA	chr8:-118620653	CCTCCAGAGCCTCACAATCC	chr8:-118620141	512	None
TAGGCAGAATCCCAACACTGAAG	chr13:-72369346	AGCATGCCTACAACACTCCC	chr13:-72369419	GGCACTAGGTCCCCTAAGGA	chr13:-72369067	352	None
CTGGGGGGCTCAAGGTTATTAGG	chr13:-29104435	GATAACCTGCTGGTAGGGAGC	chr13:-29104571	CACAACGTGGAGTAGGAGGTC	chr13:-29104238	333	None
CAGGGGCCCACAAGTTTATTGGG	chr1:+18698616	TCTATGGGTAATGGGCCAGATG	chr1:+18698447	CACATATAGGGTGGCCCAGT	chr1:+18698889	442	None
CTGGAGCCCACAAGGTTAGTCAG	chr16:+28445427	CGGATCAAGGAGCTGGCTAT	chr16:+28445244	GGACACCTTACAACGCTTGC	chr16:+28445712	468	None
CGGGAGCTCTCAAGGTTAGTCAG	chr11:+71879950	TGCCAATCTGCCCCACTTAG	chr11:+71879708	TGAGGAGCTGCAACAAGGAG	chr11:+71880138	430	None
AGAGGGCCAGCAAGGTTACTCAG	chr5:-107733643	ACGGGTAACATAGGGAAAGACAA	chr5:-107733720	GGGGGCTGCTCATATCATGG	chr5:-107733518	202	None
CGGGAGCAAGCAAGGTTCTTGAG	chr6:+144632967	AAGGCATGAAAAGTAGAAAAAGCC	chr6:+144632835	CGCCTCACACTTCCATGACA	chr6:+144633100	265	None
CAAGGGCCAGCAAGGGTATTGGG	chr4:-150681700	AACACATCCCACATCCCGAG	chr4:-150681797	CTTAAGCGGTGCTCTGGGC	chr4:-150681298	499	None
CGGCCGCCCGCAAGCTGATTGAG	chr5:+119171445	AGGGCATGAAGTGTAGTGCTC	chr5:+119171297	CACGAAAGCCGAAGGGTAGA	chr5:+119171807	510	None
CCGGGGCCCGAAAGGTCAGTGAG	chr17:+36023172	TTGAGGCTGCCTGTGAGATG	chr17:+36023040	TGTCTGCAGCATCCGAAACT	chr17:+36023492	452	None
CTGGGGCCTGCAAGGCCATTAGG	chr4:-136242201	GAACGTGTGTGAGGCAGAGA	chr4:-136242509	CTTCCGAGGTTGCATGAGGT	chr4:-136241899	610	None
TACAGCATAATCAGAGATACTGG	chr18:-4484742	TCCCACCAGTGGATTTTGTAGT	chr18:-4485010	TACACAGCCTCACGTACTGC	chr18:-4484573	437	None
AATGGCATAATCGGAGATACTGG	chr2:+137695802	GGAACTAGCCAGCCCATTGT	chr2:+137695455	TTTTCTGACCCCCACCACAC	chr2:+137696079	624	None
CACACCATAATCGTAGATACAGG	chr13:-72278554	TCAGGCCCTTAGGCTCTTCT	chr13:-72278723	GGCCTGCCAGTAGGATGTTT	chr13:-72278151	572	None
AGCAGCAAAATGGGAGATACTGG	chr2:-135266227	AATTGCTGCGCTGTAGACAT	chr2:-135266332	GATGAAGCCTCGTTGCCTTT	chr2:-135265984	348	None
TAAGGCATAATCGGAGATTCAGG	chr7:-90200445	AAAGAAAGAATGCCCCCAAG	chr7:-90200372	GGCCAGGCTACATAGCAAAC	chr7:-90199975	397	None
CATAGCACAAGGGGAGATACAAG	chr1:+75136224	CCACCATCTGCAGAGAAGCA	chr1:+75136041	GTCCTATTCGCCGACTCCAG	chr1:+75136439	398	None
CAGAGCAGAAACGGAGATAAAAG	chr17:+94412138	AACTGCGTGCAATGTGGTGA	chr17:+94411914	ATGTACTCGCATGTCCTTGCAT	chr17:+94412446	532	None
CACAACATACTTGGAGATAGAGG	chr6:-73676517	GCAGTCTCCATTTTCTCCCTGA	chr6:-73676729	ACAACCGTTCATAAGGCATCTCT	chr6:-73676189	540	None
TAGAGCATAATAGGAGATCCAAG	chrX:+45112271	TGACTCTGTTCTCGGCCATT	chrX:+45111894	ATGCTCTATTCGTGCCCTTG	chrX:+45112279	385	None
TACAGTATAAATGGAGATACGGG	chr6:+53109111	CGCAGTGTATGAGCAGGCTA	chr6:+53108863	TTTCTGGGACAGGGCAATCC	chr6:+53109475	612	None
CCTCACCCTACTGCACGCTCAAG	chr1:-94406823	TGGGAGTGATGGCTTGTGAC	chr1:-94407114	GCTTTCTCCACCCTAACCCC	chr1:-94406601	513	None
CATCACCTTGCTGCACGCCCAAG	chr2:-89400838	GGCCTTAGCGGGAAAAATAC	chr2:-89400958	ATGCCTCTCAAGGGGAGTTT	chr2:-89400577	381	None
GAACACGGTGCTGCACGCGCTGG	chr5:-115084687	CCGCACATCGTCAACTACCT	chr5:-115084758	ATGAGAGGCGAAAGGCCATC	chr5:-115084525	233	None
CGAGGCCGTGCCGCACGCTCGGG	chr4:+86503161	GAACCTTAGGGACCCAAGCC	chr4:+86502788	AACCGGCCTCTCCATAGTCT	chr4:+86503394	606	None
CTGCACCATGCGGCACGCTCCAG	chr2:-135771092	CAGGCCTTGGTTCTGGAGAG	chr2:-135771199	TCCTTTTTCCCCGTTGCCAT	chr2:-135770745	454	None
CACCCCCCTGCTGCACGCACAGG	chr13:-96588299	CATCTAGCCACCCCGTTGTT	chr13:-96588524	TCCCTCTGCTTCCTAGCACT	chr13:-96588040	484	None

CTACACCCCGCTGCAC GCTGCAG	chr1:- 54820537	GCCACCACCTTTGCTA CATCT	chr1:- 54820871	CACTTCAGAGCAGAA GAAAACCC	chr1:- 54820392	479	None
TAAAACAGTGCTGCAC TCTCAGG	chr1:+33537 592	AGGCAGGGATCTTGTA GGACT	chr1:+33537 315	ACGTTGTTCACCGCT CTGATT	chr1:+33537 827	512	None
CAAGACCCTGGGGCA CGCTCAGG	chr13:+1035 95202	GGCCTGGGTTAGGGT AAACA	chr13:+1035 94932	TGAGTAGAGGCGGTT TCCTC	chr13:+1035 95506	574	None
CATCACGGTCCTGCAC TCTCAAG	chr6:- 3328399	CTGGCTAGGGACTGG TTGTG	chr6:- 3328568	CAGGGGTAGGACAAG GAGGA	chr6:- 3328101	467	None

Table S2. Primers for RT-qPCR and constructions.

**Primers for RT-qPCR**	
mKlf4-qPCR-F1	GTGCCCCGACTAACCGTTG
mKlf4-qPCR-R1	GTCGTTGAACTCCTCGGTCT
mOct4-qPCR-F	ATCACTCACATCGCCAATCA
mOct4-qPCR-R	CCTGGGAAAGGTGTCCTGTA
mSox2-qPCR-F	ATGATGGAGACGGAGCTGAA
mSox2-qPCR-R	TTGCTGATCTCCGAGTTGTG
mNanog-qPCR-F	ACCTGAGCTATAAGCAGGTTAAGAC
mNanog-qPCR-R	GTGCTGAGCCCTTCTGAATCAGAC
mGata4-qPCR-F	CGCCGCCTGTCCGCTTCC
mGata4-qPCR-R	TTGGGCTTCCGTTTTCTGGTTTGA
mGata6-qPCR-F	TGCCTCGACCACTTGCTATGAAAA
mGata6-qPCR-R	CACTGATGCCCCTACCCCTGAG
mSox17-qPCR-F	GGAGGGTCACCACTGCTTTA
mSox17-qPCR-R	AGATGTCTGGAGGTGCTGCT
mActb(62-81)-qPCR-F	TCTTTGCAGCTCCTTCGTTG
mActb(193-212)-qPCR-R	ACGATGGAGGGGAATACAGC
**Primers for cloning**	
mKlf4-F-(EcoRI)	CG GAATTC GCCACC ATGAGGCAGCCA
mKlf4-R-(Xhol)	CCGCTCGAG AAGTGCCTCTTCATGTGTAAGGC
mKlf4-R-(Sall)	ACGCGTCGAC AAAGTGCCTCTTCATGTGTAAGGC
mKlf4-F-(BamHI)	CG GGATCC ATGAGGCAGCCACCTGGCGA
mPrmt1-F	CG GAATTCGCCACC ATGGCGGCAGCCGAGGC
mPrmt1-R-(Xhol)	CCGCTCGAG CGCATCCGGTAGTCGGTGG
mPrmt1-R-(Sall)	ACGCGTCGAC GCGCATCCGGTAGTCGGTGG
**Primers for site mutation**	
mKlf4-R387K-F	GAGCCCAAGCCAAAG**AAG**GGAAGAAGGTCGTGG
mKlf4-R387K-R	CCACGACCTTCTTCCCTTCTTTGGCTTGGGCTC
mKlf4-R389K-F	AAGCCAAAGAGGGGA**AAA**AGGTCGTGGCCCCGG
mKlf4-R389K-R	CCGGGGCCACGACCTTTTTCCCCTCTTTGGCTT
mKlf4-R390K-F	CCAAAGAGGGGAAGA**AAG**TCGTGGCCCCGGAAA
mKlf4-R390K-R	TTTCCGGGGCCACGACTTTCTTCCCCTCTTTGG
mKlf4-R394K-F	AGAAGGTCGTGGCCC**AAG**AAAAGAACAGCCACC
mKlf4-R394K-R	GGTGGCTGTTCTTTTCTTGGGCCACGACCTTCT
mKlf4-R396K-F	TCGTGGCCCCGGAAAAAAACAGCCACCCACACT
mKlf4-R396K-R	AGTGTGGGTGGCTGTTTTTTTCCGGGGCCACGA
mKlf4-R387A-F	GAGCCCAAGCCAAAG**GCG**GGAAGAAGGTCGTGG
mKlf4-R387A-R	CCACGACCTTCTTCCCGCCTTTGGCTTGGGCTC
mKlf4-R389A-F	AAGCCAAAGAGGGGA**GCA**AGGTCGTGGCCCCGG
mKlf4-R389A-R	CCGGGGCCACGACCTTGCTCCCCTCTTTGGCTT
mKlf4-R390A-F	CCAAAGAGGGGAAGA**GCG**TCGTGGCCCCGGAAA

mKlf4-R390A-R	TTTCCGGGGCCACGACGCTCTTCCCCTCTTGG
mKlf4-R394A-F	AGAAGGTCGTGGCCC**GCG**AAAAGAACAGCCACC
mKlf4-R394A-R	GGTGGCTGTTCTTTTCGCGGGCCACGACCTTCT
mKlf4-R396A-F	TCGTGGCCCCGGAAA**GCA**ACAGCCACCCACACT
mKlf4-R396A-R	AGTGTGGGTGGCTGTTGCTTTCCGGGGCCACGA
mKlf4-R394,396A-F	AGAAGGTCGTGGCCC GCGAAA GCAACAGCCACCCACACT
mKlf4-R394,396A-R	AGTGTGGGTGGCTGTTGCTTTCGCGGGCCACGACCTTCT
mKlf4-R394,396K-F	AGAAGGTCGTGGCCC AAGAAA AAGACAGCCACCCACACT
mKlf4-R394,396K-R	AGTGTGGGTGGCTGTCTTTTTCTTGGGCCACGACCTTCT
mKlf4-R394,396F-F	AGAAGGTCGTGGCCC TTCAAA TTCACAGCCACCCACACT
mKlf4-R394,396F-R	AGTGTGGGTGGCTGTGAATTTGAAGGGCCACGACCTTCT
mKlf4-R387,389K-F	GAGCCCAAGCCAAAGAAGGGAAAAAGGTCGTGGCCCCGG
mKlf4-R387,389K-R	CCGGGGCCAC GACCTTTTTC CCTTCTTTGG CTTGGGCTC
mKlf4-R389,390K-F	AAGCCAAAG**AGG**GGA**AAAAAG**TCGTGGCCC**CGG**AAA
mKlf4-R389,390K-R	TTTCCGGGGC CACGACTTTT TTCCCCTCTT TGGCTT
mKlf4-R387,389,390K-F	GAGCCCAAGCCAAAG **AAG**GGA**AAAAAG** TCGTGGCCC**CGG**AAA
mKlf4-R387,389,390K-R	TTTCCGGGGC CACGA CTTTT TTCCCTTCTT TGGCTTGGGC TC
mKlf4-R387,389,390A-F	GAGCCCAAGCCAAAG **GCG**GGA**GCAGCG** TCGTGGCCC**CGG**AAA
mKlf4-R387,389,390A-R	TTTCCGGGGC CACGA CGCTG CTCCCGCCTT TGGCTTGGGC TC
mKlf4-R387,389,390F-F	GAGCCCAAGCCAAAG TTCGGATTCTTC TCGTGGCCC**CGG**AAA
mKlf4-R387,389,390F-R	TTTCCGGGGC CACGA GAAGAA TCC GAA CTTT GGCTTGGGC TC
mKlf4-R387,389,390,394,396A-F	**GCAGCG**TCGTGGCCC**GCG**AAA**GCA**ACAGCCACCCACACT
mKlf4-R387,389,390,394,396A-R	AGTGTGGGTG GCTGTTGCTT TCGCGGGCCA CGACGCTGC
mKlf4-R387,389,390,394,396K-F	**AAAAAG**TCGTGGCCC**AAG**AAA**AAA**ACAGCCACCCACACT
mKlf4-R387,389,390,394,396K-R	AGTGTGGGTG GCTGTTTTTT TCTTGGGCCA CGACTTTTT
**Primers for Klf4 trunctions**	
mKlf4(1-157)-F	CGCGGATCCATGAGGCAGCCACCTGGCGAG
mKlf4(1-157)-R	CCGCTCGAGTTACGGGTCACCCCCGGCCCGGAT
mKlf4(1-184)-F	CGCGGATCCATGAGGCAGCCACCTGGCGAG
mKlf4(1-184)-R	CCGCTCGAGCAGGTTGAAGGGGGCCGTG
mKlf4(1-400)-F	CGCGGATCCATGAGGCAGCCACCTGGCGAG
mKlf4(1-400)-R	CCGCTCGAGTTAGTGGGTGGCTGTTCTTTT
mKlf4(1-425)-F	CGCGGATCCATGAGGCAGCCACCTGGCGAG
mKlf4(1-425)-R	CCGCTCGAGTTATGTGTGAGTTCGCAGGTG
mKlf4(158-385)-F	CGCGGATCCGGCGTGGCTGCCAGCAACACA
mKlf4(158-385)-R	CCGCTCGAGTTATGGCTTGGGCTCCTCTGGCAG
mKlf4(463-483)-F	CGCGGATCCGACCTGGCGGCGACGGTGGCC
mKlf4(463-483)-R	CCGCTCGAGTTAAAAGTGCCTCTTCATGTG
mKlf4(117-483)-F	CGCGGATCCGTGGCCGCCACCGTGACCACC
mKlf4(117-483)-R	CCGCTCGAGTTAAAAGTGCCTCTTCATGTG
mKlf4(158-483)-F	CGCGGATCCGGCGTGGCTGCCAGCAACACA
mKlf4(158-483)-R	CCGCTCGAGTTAAAAGTGCCTCTTCATGTG
mKlf4(185-483)-F	CGCGGATCCGCGGACATCAATGACGTGAGC

mKlf4(185-483)-R	CCGCTCGAGTTAAAAGTGCCTCTTCATGTG
mKlf4(386-483)-F	CGCGGATCCAAGAGGGGAAGAAGGTCGTGG
mKlf4(386-483)-R	CCGCTCGAGTTAAAAGTGCCTCTTCATGTG

Table S3. Primers for ChIP qPCR assay

	Sequence (5'-3')
Gata4 (-541/-337)-F	CGGCTCGTGGGAAGGAGAA
Gata4 (-541/-337)-R	CGGCTCGTGGGAAGGAGAA
Gata4 (+8050/+8187)-F	TCGGAGAAGAGTGAGCAG
Gata4 (+8050/+8187)-R	GTGACAGCCATAGCCAAA
Gata6 (-556/-349)-F	TCTCCGAGTTATCACAGCG
Gata6 (-556/-349)-R	GAGCAGGAAGAATGACAGG
Gata6 (+675/+820)-F	CCCATCTCGTCTTCGTCC
Gata6 (+675/+820)-R	TGCGAGGCGTAGGGGCTGA
Sox17 v1 (-219/-4)-F	CAGTTTACTTCCTGCTTCG
Sox17 v1 ((-219/-4)-R	GCTGAGAACGTCTGACCC
Sox17 v1 (+2976/+3104)-F	AGGCTAACAGTCTTACACGC
Sox17 v1 (+2976/+3104)-R	TTCCATCGCAAACACCAC
Sox17 v2 (-36/+179)-F	GGCAGGTGTAGCCAGAAC
Sox17 v2 (-36/+179)-R	CCTAACCCTTCGCCTAAT
Sox17 v2 (+724/+906)-F	ACTCAATCCCTTCTTCCAGC
Sox17 v2 (+724/+906)-R	CCTCCCTTACCTTTCTCCC
Sox7 (-245/-58)-F	ACCTGCACCGGAGCGGGATC
Sox7 (-245/-58)-R	TATCAGCTTCGGGCCACC
Sox7 (+2796/+3029)-F	ATGCCTCGTCCTCTTCAC
Sox7 (+2796/+3029)-R	TCTATTCACCATACCCTACCA
Pdgfra (-440/-273)-F	GTCCAGACAGGTGATCGGTGGTGC
Pdgfra (-440/-273)-R	GGGGCTGGGCTTCTTCCTTGGT
Pdgfra (+1216/+1319)-F	AAAGTGTTGAGGCTGCTGTGA
Pdgfra (+1216/+1319)-R	GCCCGAAGAGTTTCTGGTTTA
Amn (-1147 -892)-F	CAACCCACCAACCAAAC
Amn (-1147 -892)-R	GGAAACAGACAGTAGATGAGGC
Amn (-157/+97)-F	AGGGCAGATGCTAAAGCC
Amn (-157/+97)-R	CAGCCTCAATCAAGGTCCAG
Amn (+560 /+745)-F	GGCGACACTCAGCCTTCCT
Amn (+560 /+745)-R	CCCACCTCCAGTTCATCCC
